# Adult colocolic intussusception diagnosed by ultrasonography: a case report

**DOI:** 10.1186/1752-1947-5-294

**Published:** 2011-07-07

**Authors:** Amal Bousseaden, Rajae Afifi, Wafae Essamri, Imane Benelbarhdadi, Fatima Zahra Ajana, Moustapha Benazzouz, Abdellah Essaid

**Affiliations:** 1 Department of Gastroenterology, Medical Clinic, University Hospital Ibn Sina, Rabat, Morocco

## Abstract

**Introduction:**

Intussusception is highly uncommon in adults and accounts for only 5% of all reported cases. It is more commonly secondary to an identifiable bowel lesion in 90% of cases, whereas 10% have no discernable cause. Diagnosis is difficult due to non-specific symptoms of the disease. Diagnostic imaging plays an important role in the diagnosis of the condition. Sonography and computed tomography are the most commonly used imaging techniques. In adults, intussusception usually requires treatment by surgical resection of the affected bowel.

**Case presentation:**

A 35-year-old Moroccan woman presented with a five-month history of intermittent abdominal pain and one episode of bleeding from the rectum. At physical examination an abdominal mass was noted. Abdominal sonography revealed a 6.3 × 8.5 cm midline mass in her upper abdomen that was tender. In transverse section, the mass had the multiple concentric rings of hypoechoic and echogenic layers associated with the sonographic appearance of intussusception. In longitudinal section, the mass had the sonographic aspect of multiple parallel lines, giving the so-called "sandwich appearance".

A corresponding contrast-enhanced abdominal computed tomography scan also demonstrated the intussusception. Surgery confirmed a colocolic intussusception with a large, firm, indurated mass as the lead point. A right hemicolectomy was undertaken because of concern about possible malignancy. The resected ascending colon was then opened up, to find a protruding tumor of the ascending colon that was acting as the lead point. It measured 7.6 × 6.9 × 2.4 cm. Pathology diagnosed an infiltrating, differentiated adenocarcinoma of the ascending colon invading through the muscularis propria. No lymphovascular invasion was seen. Our patient has recovered well.

**Conclusion:**

Intussusception is relatively rare in the adult population, and this, along with the vague clinical features, makes diagnosis difficult. Ultrasonography and computed tomography have been proven to be effective diagnostic modalities. Ultrasonography can be performed quickly and accurately, and is widely available. In adults, intussusception is usually associated with an underlying cause and requires treatment by surgical resection.

## Introduction

Intussusception is highly uncommon in adults and accounts for only 5% of all reported cases. It is more commonly secondary to an identifiable bowel lesion in 90% of cases, whereas 10% have no discernable cause [1,2]. Diagnosis is difficult due to non-specific symptoms of the disease. Diagnostic imaging plays an important role in the diagnosis of the condition. Ultrasonography and computed tomography (CT) are the most commonly used imaging techniques. Adult intussusception usually requires treatment by surgical resection of the affected bowel. The aim of this paper is to report our case and to discuss the role of ultrasonography in the early diagnosis of adult intussusception.

## Case presentation

**Figure 1:**
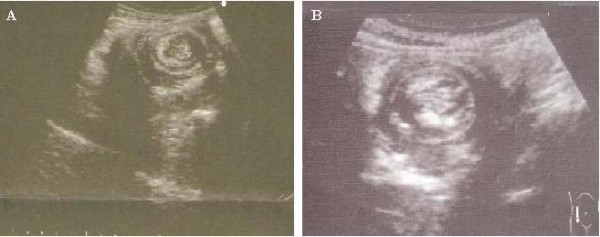
**Transverse sonographic image of the intussusception**. Note the hypoechoic outer layer of edematous bowel wall with echogenic layers, known as the "bull's-eye" or "target" signs.

**Figure 2:**
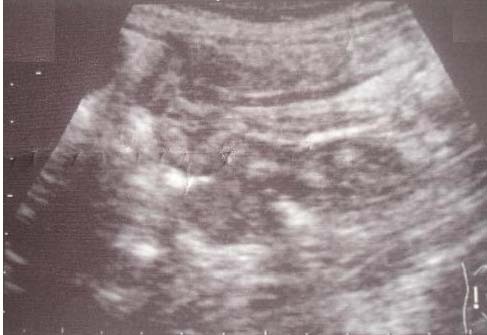
**Ultrasonographic image of intussuscepted bowel in longitudinal plane "sandwich" appearance**.

**Figure 3:**
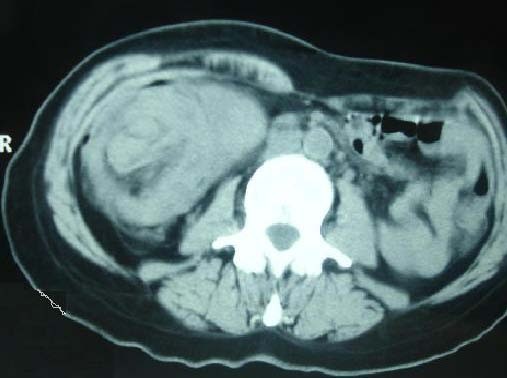
**Axial CT of the abdomen shows the edematous wall of the intussuscipiens and the mass around the invaginating mesenteric fat**.

**Figure 4:**
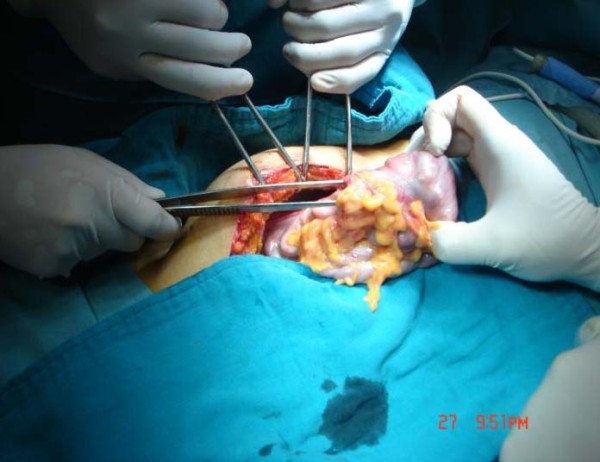
**Intussuscepted bowel prior to resection**.

**Figure 5:**
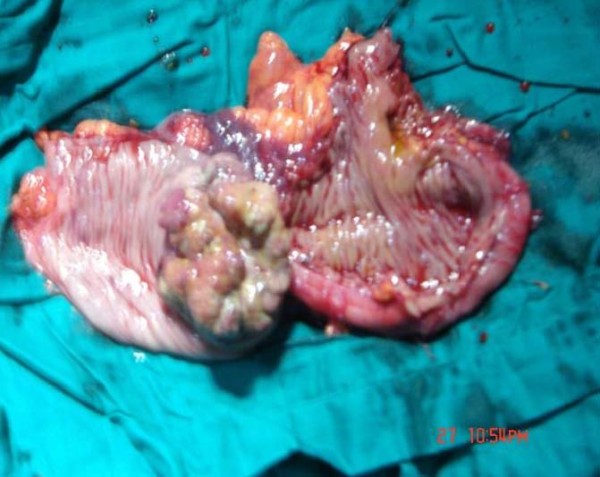
**Bowel wall is cut away, revealing the mass**.

A 35-year-old Moroccan woman presented with a five-month history of intermittent abdominal pain and one episode of bleeding from the rectum. At physical examination an abdominal mass has noted. Abdominal sonography revealed a 6.3 × 8.5 cm midline mass in her upper abdomen that was quite tender. In transverse section, the mass had the multiple concentric rings of hypoechoic and echogenic layers associated with the sonographic appearance of intussusception (Figure 1). In longitudinal section, the sonographic aspect of multiple parallel lines gave the so-called "sandwich appearance" sign as seen in Figure 2. A corresponding contrast-enhanced abdominal CT also demonstrated the intussusception (Figure 3). Surgery confirmed a colocolic intussusception with a large, firm, indurated mass as the lead point (Figure 4). Because of the concern for possible malignancy, a right hemicolectomy was undertaken. The resected ascending colon was then opened up, and a protruding tumor of the ascending colon, which was acting as the lead point, was found. The tumor measured 7.6 × 6.9 × 2.4 cm (Figure 5). Pathology diagnosed an infiltrating, differentiated adenocarcinoma of her ascending colon invading through the muscularis propria. No lymphovascular invasion was seen. She has recovered well.

## Discussion

Intussusception is relatively rare in the adult population, and this along with the vague clinical features, makes diagnosis difficult. Ultrasonography has high specificity and sensitivity, making it a valuable diagnostic tool in this adult disease, and enabling an early diagnosis.

Intussusception occurs when a segment of bowel, the intussusceptum, invaginates into the lumen of the more distal bowel, the intussuscipiens. Intussusception in adult patients may be caused by intraluminal, mural, or extraluminal lesions [3,4]. The primary mechanism by which intussusception is thought to occur is when an intraluminal mass is pulled forward by peristalsis and drags the attached bowel wall segment with it. Pedunculated tumors, such as adenomatous polyps or lipomas, are the classic examples of this group [5-7]. Most adult cases of intussusception occur in the distal small bowel (52% to 55%) or the large intestine (38% to 45%) [8]. Adult intussusception is rare and usually associated with neoplasms, of which up to 77% are malignant [9]. The most common colon cancer is primary adenocarcinoma. Leiomyosarcoma, lymphoma, and metastases as lead points have also been reported [1,10]. In our patient the colocolic intussusception was caused by an adenocarcinoma of the ascending colon. Benign colonic tumors are usually lipomas or adenomatous polyps. Inflammatory disease of the colon or appendix can represent a non-neoplastic cause of intussusception.

Adult intussusception is not easily diagnosed because patients usually present with non-specific vague symptoms such as abdominal pain, the most common symptom. Other symptoms include nausea, vomiting, and possible bleeding from the rectum [11]. Our patient presented with abdominal pain and one episode of bleeding from the rectum. Approximately 50% of patients will have had symptoms for more than one month prior to an acute exacerbation of symptoms that leads to diagnosis [12,13]. The physical findings are also non-specific and are not consistent with an acute abdomen. In our case an abdominal mass was noted. The mean age for adult intussusception is 50 years, with a nearly equal male to female ratio. Early diagnosis of intussusception may prevent the necrosis of the bowel and, in some cases, even save the patient's life [11]. As symptoms are vague, diagnostic imaging plays the main role in diagnosis. Many imaging modalities are used for diagnosis, such as radiographs, ultrasonography, CT and magnetic resonance imaging. The most commonly used are ultrasonography and CT. It is extremely important to diagnose acute intussusception as early as possible, as it leads to intestinal obstruction and cuts off the blood supply to the bowel [3,14]. Ultrasonography has high specificity and sensitivity, which makes it a valuable diagnostic tool. The classic appearance of an intussuscepted bowel on a sonographic image in a transverse plane is called the "doughnut sign" or a "target lesion" and represents several concentric rings of the bowel. Usually there is a thick hypoechoic rim with an echogenic area in the middle. The hypoechoic rim represents an edematous bowel wall, and the echogenic center corresponds to intussuscepted mesenteric fat. Sometimes, within the echogenic area in the center, an additional anechoic spot may be seen, which is believed to represent a collection of fluid in the apex of the intussusceptum [3,14]. The longitudinal appearance of intussusception usually appears as multiple parallel lines, the so-called "sandwich appearance" or "pseudo-kidney sign". The lines demonstrate bowel walls and their layers. The major limitation of ultrasonography for evaluating acute obstructive symptoms is the presence of air in the bowel, which leads to poor transmission and difficulties in image interpretation. Like sonography, CT scanning can be used to identify the intussusception; however, the underlying cause can still be difficult to determine.

In our patient the intussusception was diagnosed early with sonography and abdominal CT. Our patient went on to surgery, where the sonographic findings were confirmed. Treatment of adult intussusception is largely surgical by carcinologic resection, given the likelihood of a neoplastic etiology [4,5]. A right hemicolectomy was performed on our patient.

## Conclusion

Intussusception is relatively rare in the adult population and this, along with the vague clinical features, makes diagnosis difficult. Ultrasonography and CT have been proven to be effective diagnostic modalities. Ultrasonography can be performed quickly and accurately, and is widely available. In adults, intussusception is usually associated with an underlying cause and requires treatment by surgical resection.

## Consent

Written informed consent was obtained from the patient for publication of this case report and any accompanying images. A copy of the written consent is available for review by the Editor-in-Chief of this journal.

## Competing interests

The authors declare that they have no competing interests.

## Authors' contributions

AB cared for the patient and drafted the manuscript. AR released the ultrasonography and confirmed the intussusception diagnosis. All authors read and approved the final manuscript.

## References

[B1] Donhauser DL Kelly EC lntussusception in the adult Am J Surg 1950 79 5 673 677 10.1016/0002-9610(50)90333-3 15410943

[B2] Stuhenhord WT Thorbjarnarson B lntussusception in adults Ann Surg 1970 172 2 306 310 10.1097/00000658-197008000-00019 5433296 PMC1397058

[B3] Gayer G Apter S Hofmann C Nass S Amitai M Zissin R Hertz M Intussusception in adults: CT diagnosis Clin Radiol 1998 53 1 53 57 10.1016/S0009-9260(98)80035-4 9464437

[B4] McKay R Ileocecal intussusception in an adult: the laparoscopic approach JSLS 2006 10 2 250 253 16882431 PMC3016128

[B5] Karamercan A Kurukahvecioglu O Yilmaz TU Aygencel G Aytaç B Sare M Adult ileal intussusception: an unusual emergency condition Adv Ther 2006 23 1 163 168 10.1007/BF02850357 16644617

[B6] Reymond RD The mechanism of intussusception: a theoretical analysis of the phenomenon Br J Radiol 1972 45 529 1 7 10.1259/0007-1285-45-529-1 5008327

[B7] Huang BY Warshauer DM Adult intussusception: diagnosis and clinical relevance Radiol Clin N Am 2003 41 6 1137 1151 10.1016/S0033-8389(03)00116-7 14661662

[B8] Schuind F van Gansbeke D Ansay J Intussusception in adults Acta Chir Belg 1985 85 1 55 60 3984633

[B9] Lorenzi M Iroatulam AJ Vernillo R Banducci T Mancini S Tiribocchi A Ferrar FS Mancini S Adult colonic intussusception caused by malignant tumor of the transverse colon Am Surg 1999 65 1 11 14 9915523

[B10] Nagorney DM Sarr MG McIlrath DC Surgical management of intussusception in the adult Ann Surg 1981 193 2 230 236 10.1097/00000658-198102000-00019 7469558 PMC1345048

[B11] Reijnen HAM Joosten HJ de Boer HH Diagnosis and treatment of adult intussusception Am J Surg 1989 158 1 25 28 10.1016/0002-9610(89)90309-7 2662787

[B12] Agha FP Intussusception in adults AJR 1986 146 3 527 531 3484870 10.2214/ajr.146.3.527

[B13] Coleman MJ Hugh TB May RE Jensen MJ Intussusception in the adult Aust NZJ Surg 1981 51 2 179 180 10.1111/j.1445-2197.1981.tb05933.x 6940547

[B14] Merine D Fishman EK Jones B Siegelman SS Enteroenteric intussusception: CT findings in nine patients AJR 1987 148 6 1129 1132 3495136 10.2214/ajr.148.6.1129

